# Identification and Validation of a Prognostic 5-Protein Signature for Biochemical Recurrence Following Radical Prostatectomy for Prostate Cancer

**DOI:** 10.3389/fsurg.2021.665115

**Published:** 2021-05-31

**Authors:** Daojun Lv, Zanfeng Cao, Wenjie Li, Haige Zheng, Xiangkun Wu, Yongda Liu, Di Gu, Guohua Zeng

**Affiliations:** ^1^Guangdong Key Laboratory of Urology, Department of Urology, Minimally Invasive Surgery Center, The First Affiliated Hospital of Guangzhou Medical University, Guangzhou Medical University, Guangzhou, China; ^2^Department of Emergency Medicine, The First Affiliated Hospital of Guangzhou Medical University, Guangzhou, China; ^3^Nanshan College, Guangzhou Medical University, Guangzhou, China; ^4^Medical Imaging Center, The First Affiliated Hospital of Jinan University, Guangzhou, China

**Keywords:** biochemical recurrence, prostate cancer, prognosis, proteomic, nomogram

## Abstract

**Background:** Biochemical recurrence (BCR) is an indicator of prostate cancer (PCa)-specific recurrence and mortality. However, there is a lack of an effective prediction model that can be used to predict prognosis and to determine the optimal method of treatment for patients with BCR. Hence, the aim of this study was to construct a protein-based nomogram that could predict BCR in PCa.

**Methods:** Protein expression data of PCa patients was obtained from The Cancer Proteome Atlas (TCPA) database. Clinical data on the patients was downloaded from The Cancer Genome Atlas (TCGA) database. Lasso and Cox regression analyses were conducted to select the most significant prognostic proteins and formulate a protein signature that could predict BCR. Subsequently, Kaplan–Meier survival analysis and Cox regression analyses were conducted to evaluate the performance of the prognostic protein-based signature. Additionally, a nomogram was constructed using multivariate Cox regression analysis.

**Results:** We constructed a 5-protein-based prognostic prediction signature that could be used to identify high-risk and low-risk groups of PCa patients. The survival analysis demonstrated that patients with a higher BCR showed significantly worse survival than those with a lower BCR (*p* < 0.0001). The time-dependent receiver operating characteristic curve showed that the signature had an excellent prognostic efficiency for 1, 3, and 5-year BCR (area under curve in training set: 0.691, 0.797, 0.808 and 0.74, 0.739, 0.82 in the test set). Univariate and multivariate analyses indicated that this 5-protein signature could be used as independent prognosis marker for PCa patients. Moreover, the concordance index (C-index) confirmed the predictive value of this 5-protein signature in 3, 5, and 10-year BCR overall survival (C-index: 0.764, 95% confidence interval: 0.701–0.827). Finally, we constructed a nomogram to predict BCR of PCa.

**Conclusions:** Our study identified a 5-protein-based signature and constructed a nomogram that could reliably predict BCR. The findings might be of paramount importance for the prediction of PCa prognosis and medical decision-making.

**Subjects:** Bioinformatics, oncology, urology.

## Introduction

Prostate cancer (PCa) is the second leading cause of tumor death among American males and accounts for 20% of newly-diagnosed cancers, with 31,620 deaths reported in 2019 (1). Although radical prostatectomy (RP) is considered as an effective method of therapy for PCa patients, recent studies have revealed that ~20–40% of patients suffer from biochemical recurrence (BCR) after RP (2). BCR is characterized by a recurrent prostate specific antigen (PSA) concentration of more than 0.2 μg/L and is an indicator for distant metastasis or PCa-specific mortality (2, 3). 32–45% of BCR patients with post-RP are predicted to die from PCa within 15 years (4). Only patients with high-risk (Gleason score of 8–10 or a PSA doubling time of 12 months) will benefit from salvage treatment (5). Thus, early identification of PCa patients with high BCR risk has great importance for choosing the best management strategy.

The 2014 version of the Gleason grading system (6), PSA, and tumor stage are currently recommended to be used to predict BCR, based on their association with the survival of PCa patients (7). However, prediction based on clinicopathological features has limited accuracy (8). Patients with similar clinicopathological features may reach infinitely different clinical endpoints (9). It has been well-acknowledged that although biochemical processes from DNA to protein are influenced by many complicated biological factors, proteins can be used to directly determine the functions of genes. Proteins are the main executors of life processes, and changes in gene function are usually accompanied by changes in protein expression, modification, or stability. Moreover, there may or may not be a correlation between the protein and mRNA expression levels of a particular gene (10). The quantification of proteins may be indicators for aggressiveness of tumors (11). To date an increasing number of nuanced BCR risk stratification systems have been developed as gene biomarkers (12), while little emphasis has been placed on the potential function of protein-based signatures for the prediction of BCR in PCa.

In this study, to predict the BCR of PCa patients, we proposed to (1) construct a multi-protein-based signature and nomogram that combined clinicopathological variables to predict the prognosis of BCR for PCa patients, (2) validate predictive ability using time-dependent receiver operating characteristic (ROC) curves, calibration plots, concordance index (C-index), and decision curve analysis (DCA), (3) perform GO (Gene Ontology) pathway enrichment analyses and Gene Set Enrichment Analysis (GSEA) to investigate biological functions that are involved.

## Methods

### Collection of Protein and Clinical Data

The preset endpoint of our work was BCR after RP. Data acquisition and application were conducted based on publication policies and guidelines for the protection of human subjects.

Reverse-phase protein array (RPPA) protein data on prostate adenocarcinoma after RP was searched for and obtained from the Cancer Proteome Atlas (TCPA) database (13). The Cancer Genome Atlas (TCGA) database was used to download clinical data and RNA-sequencing data of the PCa samples. After analyzing the data downloaded, patients without data integrality, as indicated by the following features, were eliminated: (1) a follow-up period of <30 days, and (2) a lack of clinical information, such as recurrence and TNM stage, or demographic data, such as age. Then, the patients were randomly assigned to the training and internal validation set by a ratio of 1:1.

### Identification and Validation of the Multitype-Protein-Based Prognostic Signature

Univariate Cox regression analysis was applied to identify the prognostic protein signature in the training set. To construct prognostic protein signature, only proteins with *P* < 0.05 were considered to be significantly prognostic factors and were used as candidate proteins for further model construction. Log-rank tests and Kaplan–Meier (KM) survival analyses were subsequently conducted to investigate the prognostic ability of the proteins identified through the univariate Cox regression analysis. Using the “glmnet” package in R, least absolute shrinkage and selection operator (Lasso)-penalized cox regression analyses were conducted to further narrow down proteins that could be used to predict BCR in the training set. Regression coefficients and coefficients of other unrelated variables were set to zero using Lasso in terms of the regulation weight, λ (14). The best λ value was obtained based on the minimum cross-validation mistake using 10-fold cross-validation. A list of prognostic proteins with related coefficients were screened for based on the optimal λ value. Finally, protein signature was constructed by a multivariable Cox regression model based on the result from Lasso analysis, and the risk score of each patient was calculated by weighted estimators corresponding to the expression level of protein.

To investigate the prognostic accuracy of the protein signature, hierarchical clustering analysis was conducted to classify the data in the training dataset using the “heatmap” R package. The median risk score was used as cut-off score to identify high- and low-risk patients. To further prove the discriminatory power of the protein-signature, we conducted a KM analysis with log-rank test and ROC curve. To evaluate the potential and applicability of protein signature, the validation was done in the internal validation and entire cohorts.

Univariate and multivariate Cox regression analysis was conducted in entire cohort to explore whether this protein-base-signature was independent of age, Gleason score, T stage, N status, PSA, and residual tumor. KM curves were applied to investigate the prognostic value of the protein signature in different clinical features. Additionally, the area under the curve (AUC) of the time-dependent ROC curve was calculated to compare the prognostic performance between the protein signature and clinical variables.

### Construction and Validation of a Predictive Nomogram

All independent prognostic proteins and the relevant clinical data were used to construct a prognostic nomogram by conducting a multivariate Cox regression analysis on the entire set. The stepwise method was applied to select the most suitable model, and a risk score was assigned to the expression level of each prognostic protein and coefficients were weighted throughout the Cox model.

To investigate the predictive accuracy of the nomogram, the signature and clinicopathological factors, including age, Gleason score, T stage, N status, PSA and residual tumor, the AUC of the ROC was determined. Calibration plots were used to further assess the discriminative power of the nomogram. The 45° line represents the best prediction. Subsequently, the C-index was determined to explore the predicted probabilities of the nomogram. Finally, a decision curve analysis (DCA) was used to investigate the clinical net benefit of the diverse probability thresholds for possible clinical outcomes and reliability of the nomogram (15).

### Functional Enrichment Analysis of Prognosis Proteins

We calculated the *Pearson's* correlation coefficient between the level of protein expression from the signature and expression of genes. Then, GO pathway enrichment analyses were performed to identify potential biological function of prognosis proteins. A *P* < 0.05 was considered as the cut-off criteria. GSEA was conducted using GSEA software on JAVA platform to investigate potential biological functions to identify proteins to be included on the signature (16). FDR < 0.05 and *P* < 0.05 were preset as the cut-off criteria to identify the enriched group. A Sankey diagram was constructed using the “ggplot2” package in R to identify co-expression proteins.

### Statistical Analysis

All statistical analyses were conducted using R 3.5.0 software. After calculating the risk value for each patient based on the regression coefficient of each protein in the risk model, the PCa patients were randomly assigned into either the high-risk or low-risk group based on the median risk score, using the “caret” package of R (set. Seed = 1,000, *p* = 0.50). The regression coefficients of the five prognostic proteins were obtained from the multivariate Cox proportional hazards regression model. We then utilized a linear combination method to determine a risk score formula, as follows:

$$\text{Risk score=}\sum_{\text{n=1}}^{\text{5}}\text{Exp*}\delta ;$$

where, Exp is the expression level of each prognostic gene, and δ is the regression coefficient of it.

A prognostic signature was constructed using univariate Cox regression analysis and the “Survival” package in R. To evaluate the prognostic power of the multi-protein-based signature, a time-dependent receiver operating characteristic (ROC) analysis was conducted and the concordance index (C-index) was calculated based on the “survival ROC” R package. BCR, was calculated from the day of RP to the time of recurrence, in terms of the KM model, and the log-rank test was used to evaluate statistical differences between the high-risk and low-risk groups. A multivariate Cox regression model was used to construct a nomogram using the “rms” package. The hazard ratios (HR) and 95% confidence intervals were calculated to identify proteins associated with BCR. *P* < 0.05 was considered statistically significant.

## Results

### Characteristics of the Patients

After samples without adequate clinical information were removed, a total of 341 patients from TCGA were included in this study. RP was conducted on all Patients and they were biochemically diagnosed with BCR. Then, the patients were assigned to either the training set (*n* = 169) or the testing set (*n* = 172), respectively. The mean age of the patients was 61.5 years (standard deviation = 6.65) and the mean follow-up duration was 2.68 years (standard deviation = 2.02). Moreover, 30 out of 341 patients had low Gleason score (≤6) PCa, while 140 patients had high Gleason score (≥8) PCa.

Prognostic proteins were screened for by conducting a univariate Cox regression analysis to identify proteins associated with BCR. As a result, a total of 21 proteins associated with BCR were identified. KM analyses were conducted to verify the identity of the 21 selected proteins (*p* < 0.05; Supplementary Figure 1).

### Development and Validation of the Proteins Signature

**Figure 1:**
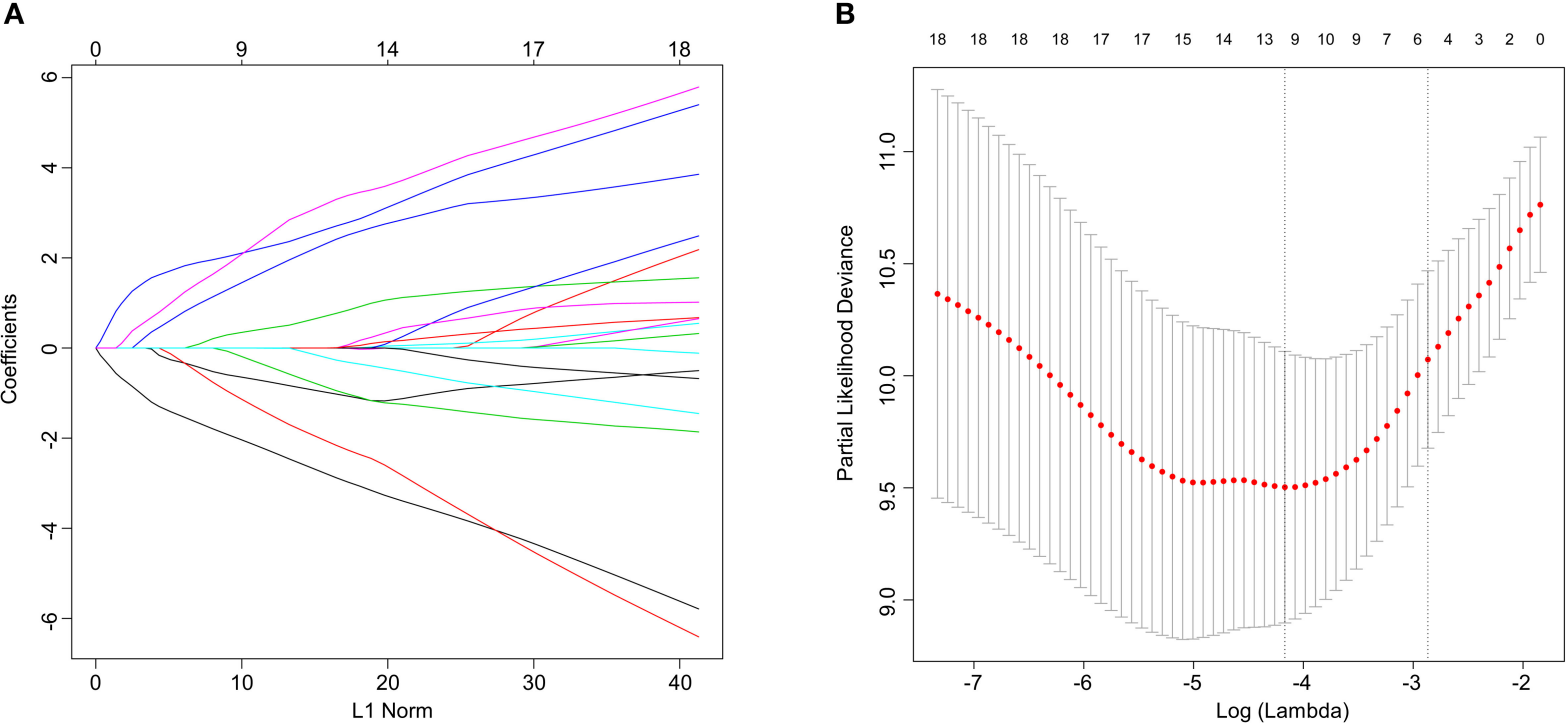
*Lasso* Cox regression analysis. **(A)** Ten-time cross-validation for tuning parameter selection in the lasso model. **(B)** *Lasso* coefficient profiles of the 21 predictive proteins associated with BCR. A vertical line is drawn at the value selected by 10-fold cross-validation.

Cox regression analysis was conducted to construct a prognostic model in the training set, which identified 5 proteins (*alpha-Catenin, BRD4, DJ1, SMAD1*, and *YB1*) from among the 21 proteins initially identified (Figure 1). An equation to calculate the BCR risk score was derived based on the expression levels of the five proteins weighted by the regression coefficients. The equation was: risk score = (−2.771 × levels of *alpha-Catenin*) + (1.577 × levels of *BRD4*) + (−2.239 × levels of *DJ1*) + (2.152 × levels of *SMAD1*) + (2.428 × levels of *YB1*). Among these five prognostic proteins, three (*BRD4, SMAD1, YB1*) demonstrated positive coefficients, suggesting that elevated expression levels of these proteins were associated with a high-risk of BCR. Two (*alpha-Catenin* and *DJ1*) of the proteins included in the Cox regression analysis were found to have a negative coefficient, indicating that elevated expression levels of these proteins were related with better survival.

**Figure 2:**
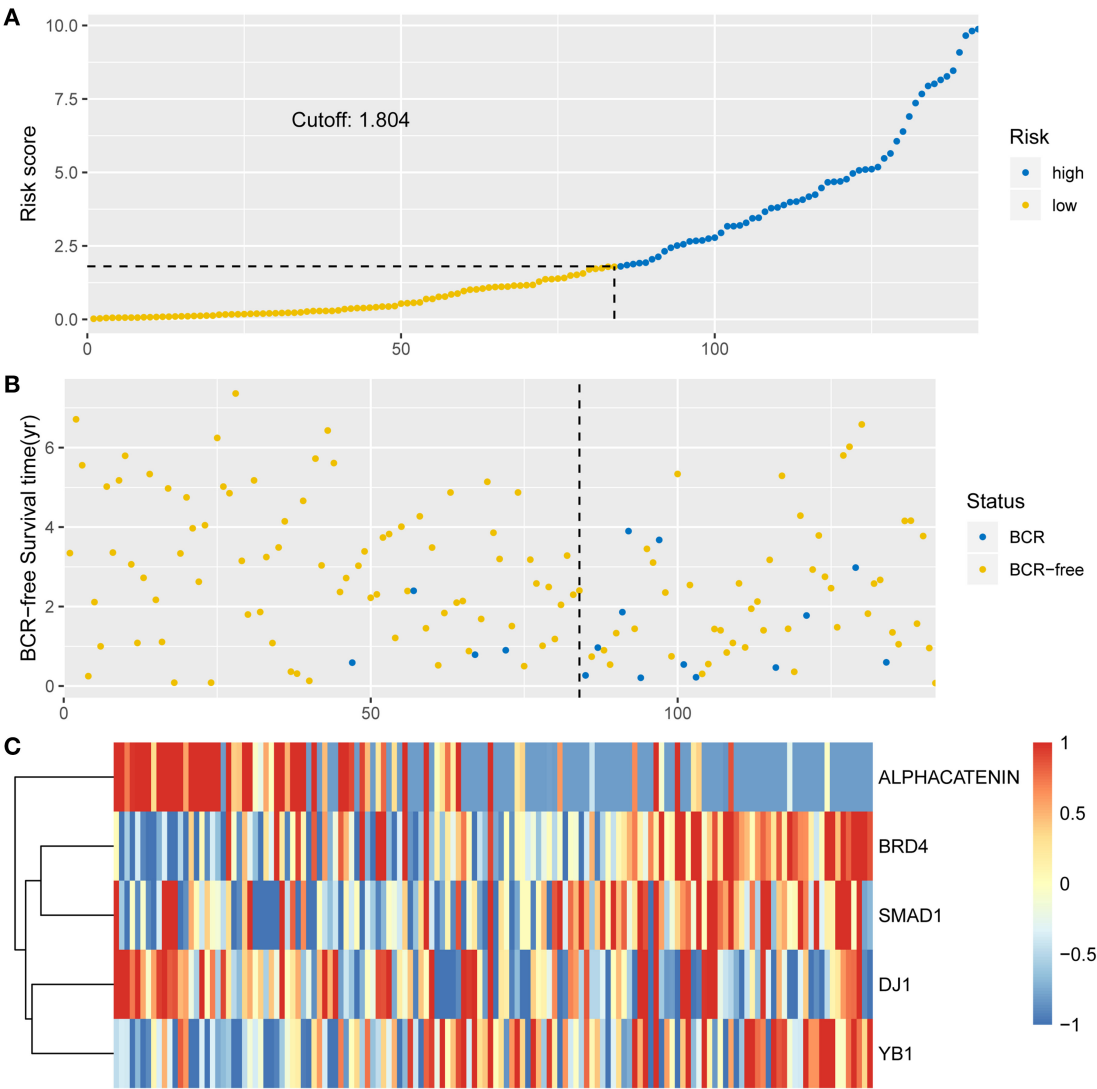
Identification of the integrated prognostic classifier in the training set. **(A)** The distribution of risk score. The median risk score cut-off is 1.804. **(B)** Each point in the scatterplot represents the survival status of patients. **(C)** Heat map showed differentially expressed proteins between BCR-free patients and patients with BCR.

To investigate the predictive performance of the signature, patients were assigned into high-risk and low-risk groups based on the median risk score. PCa patients with a risk score of 1.804 or lower were assigned to the low-risk group, while others were assigned to the high-risk group (Figure 2A). For BCR, a higher risk score indicated worse prognosis. Therefore, compared with the low-risk group, patient death was significantly higher in the high-risk group (Figure 2B). The expression levels of proteins with a positive coefficient were higher in the high-risk group (Figure 2C). We also found that patients with a high BCR risk were more inclined to express the high-risk proteins, whereas samples with low BCR tended to express protective proteins more often.

**Figure 3:**
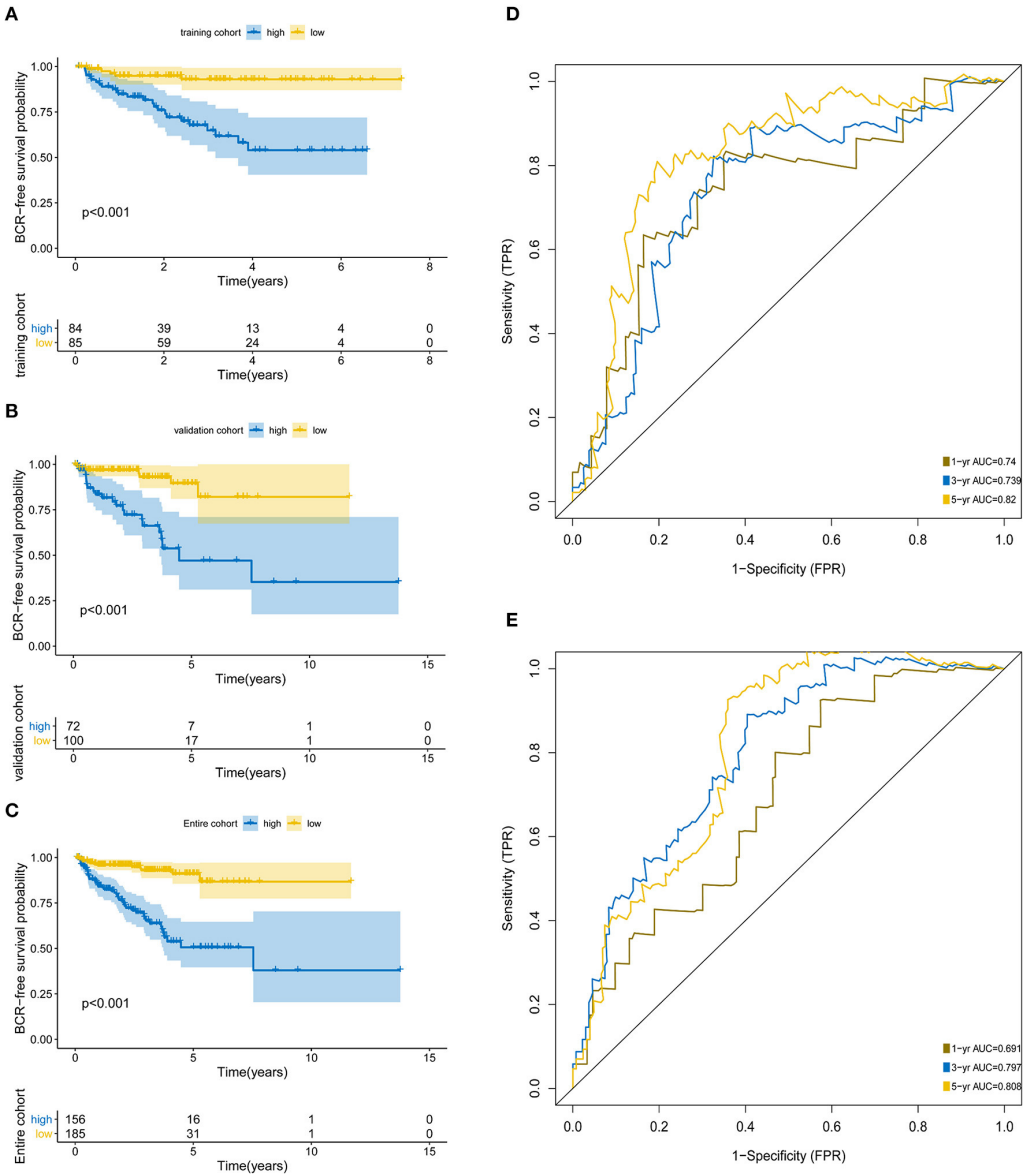
Kaplan-Meier survival analysis and ROC curves according to the prognostic signature. Kaplan–Meier curves for **(A)** the training group (*N* = 172); **(B)** the test group (*N* = 169); **(C)** the entire group (*N* = 341). ROC curves at 1, 3, 5 years for **(D)** training group, **(E)** test group.

The KM analysis demonstrated that patients with higher BCR showed significantly worse survival than those with lower BCR (*p* < 0.0001; Figures 3A–C). The AUCs of the 5-protein-based signature at 1, 3, and 5-year survival were 0.691, 0.797, 0.808 for the training set, and 0.74, 0.739, 0.82 for the test set, indicating that the prognostic signature showed a high level of specificity and sensitivity (Figures 3D,E).

**Figure 4:**
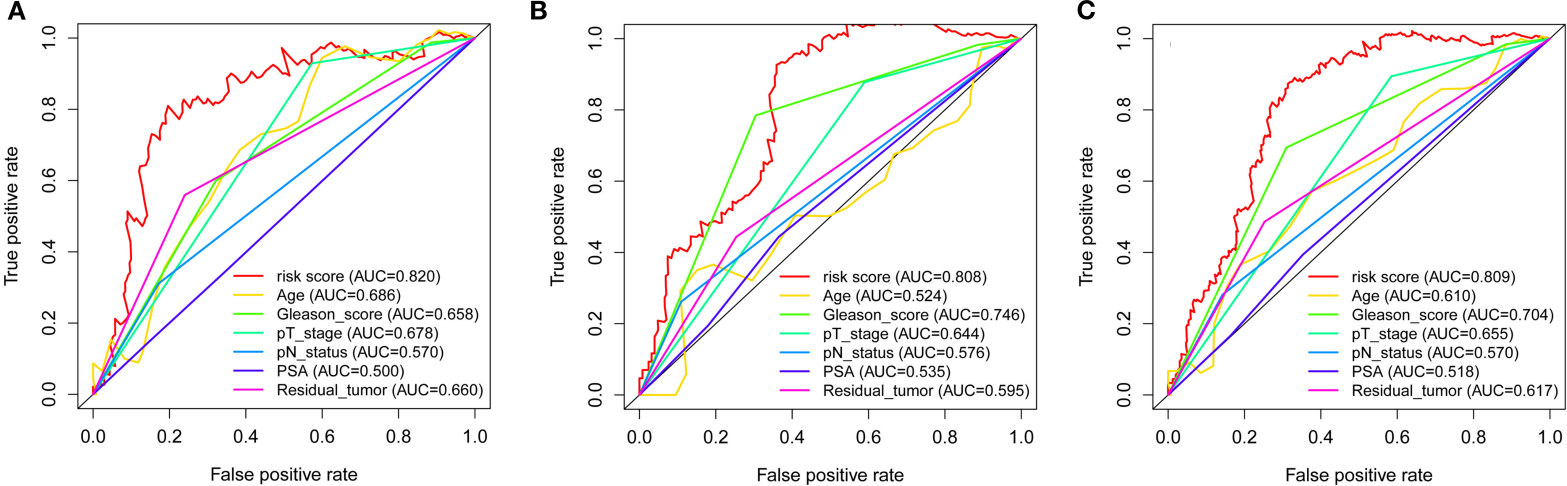
ROC curves compare the prognostic power between the prognostic signature and clinicopathological features. **(A)** Training set. **(B)** Test set. **(C)** Entire set. *P*-values indicate the area under curve (AUC) at 5 years for multi-protein-based signature verse the AUC at 5 years for other features.

Throughout the univariate and multivariate Cox proportional hazards regression analyses, the 5-protein predictive signature was confirmed to be independent of other clinicopathological factors, including age, Gleason grades, T stage, N status, PSA, and residual tumors in predicting BCR-free survival (Table 1). Moreover, the AUC of the ROC showed that the 5-protein-based signature showed significantly better prognostic performance than other clinical factors in the training, test and the set as a whole (Figure 4). The KM survival analysis also confirmed the discriminative capability of the signature under different clinical prognosis related features (Supplementary Figure 2).

**Table 1:** Univariate and multivariate analysis of different prognostic parameters in patients with prostate cancer by cox regression analysis.

**Variable**	**Univariate analysis**		**Multivariate analysis**	
	**HR (95% CI)**	***P*-value**	**HR (95% CI)**	***P*-value**
**Training dataset (*****n*** **=** **169)**				
Age	1.005 (0.949–1.064)	0.872	0.960 (0.899–1.026)	0.228
Gleason scores	5.205 (2.308–11.738)	<0.001	3.138 (1.194–8.244)	0.020
T stage	4.64 (1.407–15.298)	0.012	2.903 (0.823–10.242)	0.097
N status	2.683 (1.193–6.034)	0.017	1.425 (0.600–3.385)	0.422
PSA	1.175 (0.760–1.818)	0.468	0.719 (0.442–1.171)	0.185
Residual tumor	2.144 (1.040–4.422)	0.039	1.301 (0.593–2.855)	0.512
Risk score	8.212 (2.859–23.592)	<0.001	4.240 (1.381–13.016)	0.012
**Test dataset (*****n*** **=** **172)**				
Age	1.060 (1.003–1.120)	0.040	1.047 (0.984–1.113)	0.146
Gleason scores	2.725 (1.355–5.480)	0.005	1.368 (0.596–3.143)	0.460
T stage	5.437 (1.584–18.665)	0.007	3.041 (0.759–12.187)	0.116
N status	2.236 (1.032–4.846)	0.041	1.003 (0.412–2.440)	0.995
PSA	1.105 (0.687–1.777)	0.681	1.022 (0.590–1.770)	0.939
Residual tumor	2.326 (1.102–4.911)	0.027	1.131 (0.491–2.601)	0.773
Risk score	5.704 (2.423–13.426)	<0.001	4.401 (1.818–10.653)	0.001
**Entire dataset (*****n*** **=** **341)**				
Age	1.033 (0.993–1.075)	0.109	1.006 (0.963–1.051)	0.783
Gleason scores	3.702 (2.190–6.258)	<0.001	1.890 (1.043–3.421)	0.036
T stage	4.889 (2.073–11.530)	<0.001	2.611 (1.049–6.508)	0.040
N status	2.355 (1.350–4.109)	0.002	1.160 (0.634–2.122)	0.629
PSA	1.148 (0.833–1.581)	0.399	0.852 (0.593–1.225)	0.388
Residual tumor	2.156 (1.286–3.616)	0.004	1.283 (0.730–2.258)	0.387
Risk score	6.660 (3.450–12.859)	<0.001	4.653 (2.358–9.181)	<0.001

*CI, confidence interval; HR: Hazard Ratio*.

### Identification and Validation of the Nomogram

**Figure 5:**
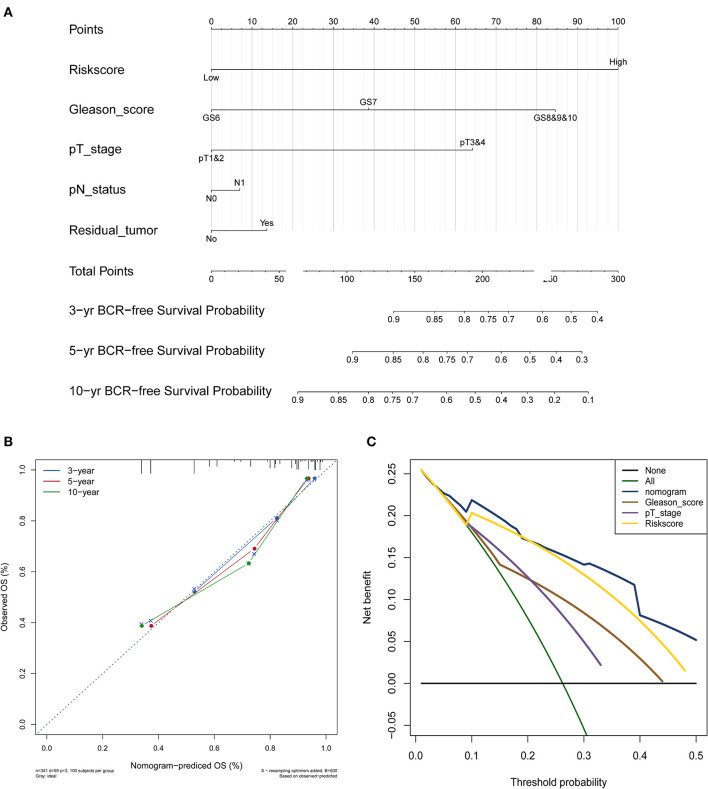
PCa survival nomogram, calibration curve, and Net benefit curves. **(A)** Survival nomogram. each variable axis represents the value of individual patient, and a line is plotted upward to decide the number of points received for each variable value. The Total Points axis represents the total of these numbers, and a line is plotted downward to the survival axes to deicide the probability of 3-, 5-, and 10-year BCR-free survival. **(B)** The calibration curve for predicting PCa patient survival at 3, 5, and 10 years in the entire cohort. **(C)** Net benefit curves for the nomogram, signature, and other clinical features, along with their confidence intervals. *P*-values indicate the area under curve (AUC) at 5 years for nomogram verse the AUC at 5 years for other features.

The nomogram was constructed based on the entire set using the multivariate Cox regression analysis of the 5 proteins against preset clinicopathological covariables. The results demonstrated the good prognostic performance of BCR in the PCa patients (Figure 5A). Calibration plots confirmed the predictive value of the prognostic nomogram for 3, 5, and 10-year BCR overall survival (OS) (Figure 5B). The C-index of the nomogram was 0.777 (95% CI: 0.699–0.855) in the training set, 0.771 (95% CI: 0.691–0.851) in the test set, and 0.764 (95% CI: 0.701–0.827) in the entire set. The net benefit curves showed that the nomogram had a superior prediction ability than the signature and other clinicopathological factors (Figure 5C).

### Functional Characteristics of the 5 Proteins

**Figure 6:**
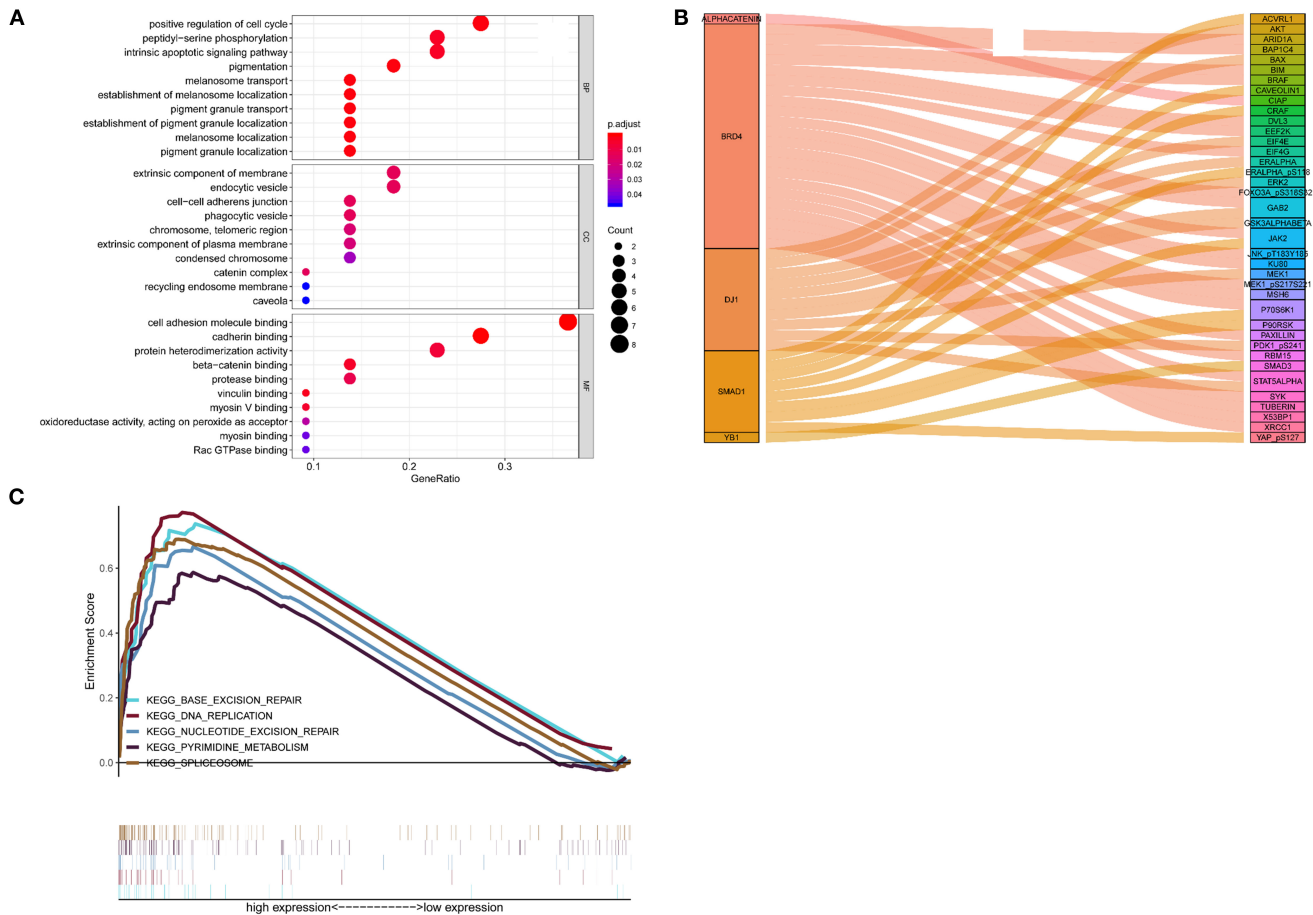
Functional characteristics of 5 proteins. **(A)** GO enrichment analysis, **(B)** Gene set enrichment analysis, **(C)** Sankey diagram of the signature.

Positive correlations between the 5 proteins and their corresponding genes were determined by calculating the Pearson correlation coefficient shown in Supplementary Figure 3. The outcomes of the GO enrichment analysis indicated that these protein-related genes were enriched in immune or cell differentiation-related GO terms (Figure 6A), suggesting that the effect of these prognostic proteins may be associated with the tumor microenvironment. Sankey diagram was used to visual the relation among the 5 proteins and other proteins in TCGA set (Figure 6B). Additionally, GSEA was conducted using TCGA database to ascertain the five proteins associated with biological signaling pathway between the high-risk and low-risk groups (Figure 6C). The following five pathways were identified: (1) base excision repair, (2) DNA replication, (3) nucleotide excision repair, (4) pyrimidine metabolism, and (5) spliceosome. Finally, the Sankey diagram revealed the association between the co-expression proteins and 5-protein signature, which may interact with each other through certain molecular mechanisms (Figure 6C).

## Discussion

Most patients in the early stages of BCR develop clinical recurrence and require timely intervention (2). Up to date, certain novel biomarkers of BCR in PCa have been identified (7, 12, 17). However, most of these studies focused on only one or a few genes, and very little work was carried out on the clinical predictive performance of the proteins. The clinical applicability of the mechanisms used were also restricted due to the high cost involved (14). Due to the heterogeneity of PCa (18), a protein-base-panel of biomarkers may be more sensitive and specific to predict the prognosis of malignant prostate disease compared with a single gene. Notably, tumor protein biomarkers are reliable, economical, fast, and easily measurable (19, 20), therefore they are more suitable for use at health centers (20). To date, protein models have demonstrated their important role in predicting the prognosis of several tumors, such as bladder cancer and esophageal squamous cell carcinoma (21, 22). The abnormal expression of protein biomarkers in tumor cells may indicate the prediction potential. To the best of our knowledge, this is the first study that has constructed a prognosis protein-based model for patients with PCa after RP.

In this present study, we established and validated the 5-protein-based signature (*alpha-Catenin, BRD4, DJ1, SMAD1*, and *YB1*) to predict the prognosis of BCR in patients with PCa. Initial concerns on the poor value of the proteins is not supported (23) due to significant correlations between gene and protein expression levels observed in this study. The results of the KM curves and the C-index revealed that the 5-protein-based signature may be of importance for categorizing patients into high-risk and low-risk BCR groups, as an effective indicator of prognostic. Shao et al. (7) established a 5-lncRNA-based signature to predict PCa BCR, which resulted in an AUC of 0.68. However, our 5-protein-based signature showed better clinical utility, outperforming the known model with an AUC of 0.809 in the complete cohort, 0.808 in the test cohort, and 0.820 in the training cohort. Additionally, the current TNM staging system was closely associated with the prognosis of PCa. Consistently, in the present study univariable and multivariable Cox regression analyses also showed that tumor TN stage was a significant prognostic factor for PCa. It is worthy to note that our 5-protein signature was found to be independent of tumor stage throughout the KM analysis, indicating its ability to differentiate PCa patients with high BCR risk.

Furthermore, Gerke et al. constructed a four-protein cancer nomogram that included *PTEN, SPP1, SMAD4*, and *CCND1*, which are associated with lethal outcomes among PCa patients, using clinical features (24). However, their nomogram failed to provide long-term and independent predictive information beyond clinical factors. In contrast, our 5-protein–based nomogram has greater discriminatory ability, compared with other clinicopathological features. Moreover, calibration plots and DCA showed great predictive performance in predicting the 3, 5, and 10-year BCR overall survival. Overestimating the risk of indolent PCa patients, who are more suitable for active surveillance, results in a higher public health burden due to overtreatment (7). Thereby, our nomogram with strong discriminative power could provide new insights on the appropriate method of treatment for the better clinical management of PCa patient cohorts.

Given the considerable value of determining the risk of BCR, the potential predictive performance of the individual proteins included in our final five-protein-signature needed to be revealed. Our study revealed that three proteins (*BRD4, YB1*, and *SMAD1*) were risk factors for PCa patients. *BRD4* is homologous to the murine protein *MCAP*, which is associated with chromosomes during mitosis, and to the human RING3 protein, a serine/threonine kinase. Tan et al. (25) reported that *BRD4* was significantly elevated in malignant prostate specimens and is associated with the clinical stage and metastasis. Further analysis demonstrated that *BRD4* may even mediate the migration and invasion of castration-resistance prostate cancer (CRPC) through direct transcriptional regulation (26). Accordingly, we found that an elevated *BRD4* expression level was associated with poor overall survival in PCa. *YB-1* is known to be translated from *YBX1*, which regard as a transcription factor. *YB-1* has also been revealed to drive tumorigenicity and the invasiveness of PCa and is correlated with a poor outcome in CRPC. Around 66% of patients with high *YB-1* expression were reported to relapse within 5 years of post-operative chemotherapy (27). The potential molecular mechanisms that result in the upregulation of *YB-1* may contribute to decreased intracellular androgen accumulation, thus weaning PCa off androgen dependency and upregulating tumor survival (28). Our findings were consistent with the results of previously studies, which identified *YB1* protein as a risk factor for BCR in PCa. Smad proteins are regarded as central modulators of *TGF-*β and *BMP* signaling pathways, which regulate malignant cell growth and differentiation. *SMAD1* is a *Smad* protein. Evidence obtained from recent studies has suggested that *SMAD1* is notably elevated in patients with high-risk PCa (29). The same conclusion is also drawn by increasing evidence, which showed that the downregulation of *SMAD1* contributed to PCa proliferation, migration, and invasion (30). In our study, we found that *SMAD1* functioned as a tumor promoter, which may have partially contributed to the progression of BCR. However, only a limited number of studies have focused on the association between *SMAD1* and PCa. Therefore, further studies are necessary to obtain compelling evidence to fully understand the mechanisms involved.

In this study, we also revealed two (*alpha-Catenin* and *DJ1*) proteins that function as protective factors against PCa progression. *Alpha-Catenin* is a cadherin-associated protein that functions as an important cell adhesion molecule. It has been reported that changes in *alpha-catenin* regulate the cell-cell adhesion mechanism, which seems to be present in almost half of all prostate tumors (31). Furthermore, *alpha-catenin* has been proven to be a promising prognostic marker for PCa specific survival, and a lack of it may indicate PSA failure, as well as shortened survival (31, 32). Recent literature has demonstrated that a lack of *alpha-catenin* expression leads to decreased cell-cell adhesion and loss of the epithelial phenotype, which can be reversed after repletion of *alpha-catenin* (33). *DJ-1* is translated from the *PARK7* gene, which belongs to the peptidase C56 family of proteins. It acts as a redox-sensitive chaperone and as a sensor for oxidative stress, which protects neurons against oxidative stress and cell death. Although Xu et al. (34) has indicated that *DJ-1*/*PARK7* may function as a positive regulator of androgen receptor-dependent transcription and its ability to differentiate between PCa patients and healthy individuals has also been demonstrated. Our results indicate that *DJ-1* might be a protective factor against PCa progression. However, future prospective studies need to be conducted to determine the significance of *DJ-1/PARK7* in PCa progression to identify the underlying mechanisms.

The signature constructed in this study was derived from BCR-related proteins. Therefore, these signatures will also be appropriate for the prognostic evaluation of PCa. Furthermore, the pathway analysis also confirmed that our 5-protein signature is closely associated with cancer metastasis and BCR. Interestingly, the Sankey diagram indicated potential interactions between the five proteins included in our signature and co-expressed genes. The findings of our study are consistent with the study conducted by Augustin et al. (35), who suggested that prostate tumor grade is associated with the expressions of *p53* and alpha-catenin. Mutations of the *p53* gene and depletion of *alpha-catenin* are observed in pats of PCa (35). In addition, the Sankey diagram constructed in this study broadly supported the work of Gulino-Debrac (36), who indicated that *alpha-catenin* and *VEGFR2* may be associated with the mechano-transduction mechanism of adherent junction strengthening of endothelial cell-cell contacts. Since most co-expression proteins in PCa have not yet been functionally annotated, future studies should be conducted to determine the potential molecular connections involved in PCa progression.

Although the protein-base model showed an accurate survival prognosis, there are several limitations in our study. First, only patients with complete BCR information were included in this study, which may have created a selection bias. The sample size of patients included in this study was also limited. Secondly, the potential molecular mechanisms of several proteins in our signature have not been completely elucidated. Therefore, further research needs to be conducted using larger sample sizes to explore the precise molecular mechanisms involved with these prognostic proteins. Third, the AUC and C-index did not exceed 0.8 in our study, the results were better than that of lncRNA–based nomograms (AUC = 0.68 at 2 years; C-index = 0.74) that were designed to predict BCR-free survival in PCa (7). Further studies that test the practical applicability of our signature in clinical practice need to be conducted.

## Conclusions

To predict biochemical recurrence following radical prostatectomy in PCa, we first constructed and validated an innovative prognostic proteomic signature, which could stratify PCa patients into a high-risk group and low-risk group. Moreover, the nomogram contained more prognostic parameters than traditional staging systems, indicating the discriminative power and promoting the personalized management of PCa patients. This was particularly obvious for those with Gleason scores or PSA levels that did not match the clinical endpoints. Therefore, more large-scale, prospective, multi-center trials are necessary to verify our results before this protein signature can be applied to a clinical setting.

## Data Availability Statement

The datasets presented in this study can be found in online repositories. The names of the repository/repositories and accession number(s) can be found in the article/Supplementary Material.

## Author Contributions

DL, DG, and GZ: conception and design. DL, HZ, XW, and WL: collection and assembly of data. DL, WL, and HZ: data analysis and interpretation. ZC, WL, DL, XW, and YL: writing, review, and/or revision of the manuscript. YL and GZ: administrative, technical, or material support. YL, DG, and GZ: study supervision. All authors have given final approval of the version to be published and agreed on the journal to which the article has been submitted.

## Conflict of Interest

The authors declare that the research was conducted in the absence of any commercial or financial relationships that could be construed as a potential conflict of interest.

## Abbreviations

BCR: biochemical recurrence
PCa: prostate cancer
RP: radical prostatectomy
CRPC: castration-resistance prostate cancer
ROC: receiver operating characteristic
C-index: concordance index
DCA: decision curve analysis
GO: Gene Ontology
GSEA: Gene Set Enrichment Analysis
RPPA: Reverse-phase protein array
TCPA: Cancer Proteome Atlas
TCGA: Cancer Genome Atlas
FPKM: Fragments Per Kilobase Of Exon Per Million Fragments Mapped
KM: Kaplan–Meier
Lasso: least absolute shrinkage and selection operator
AUC: area under curve
DCA: decision curve analysis
MSigDB: Molecular Signatures Database
HR: hazard ratios.
