# Supplementary figures and images for: Identification and Validation of a Prognostic 5-Protein Signature for Biochemical Recurrence Following Radical Prostatectomy for Prostate Cancer
Source: Front Surg. 2021 May 31;8:665115. doi: 10.3389/fsurg.2021.665115 (PMC8202683; doi:10.3389/fsurg.2021.665115)

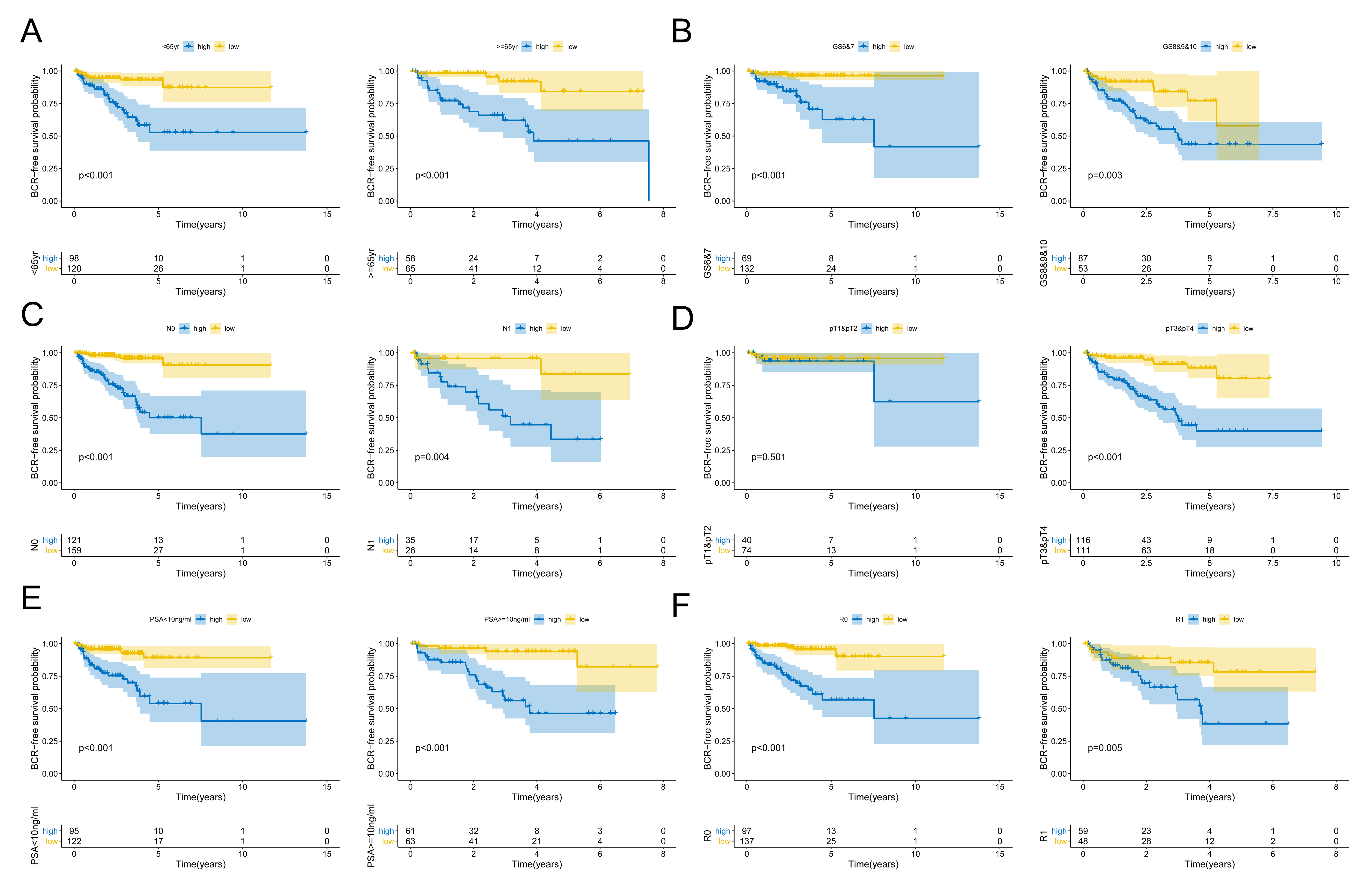

Supplement: Supplementary file 1 [file Image_1.TIF]

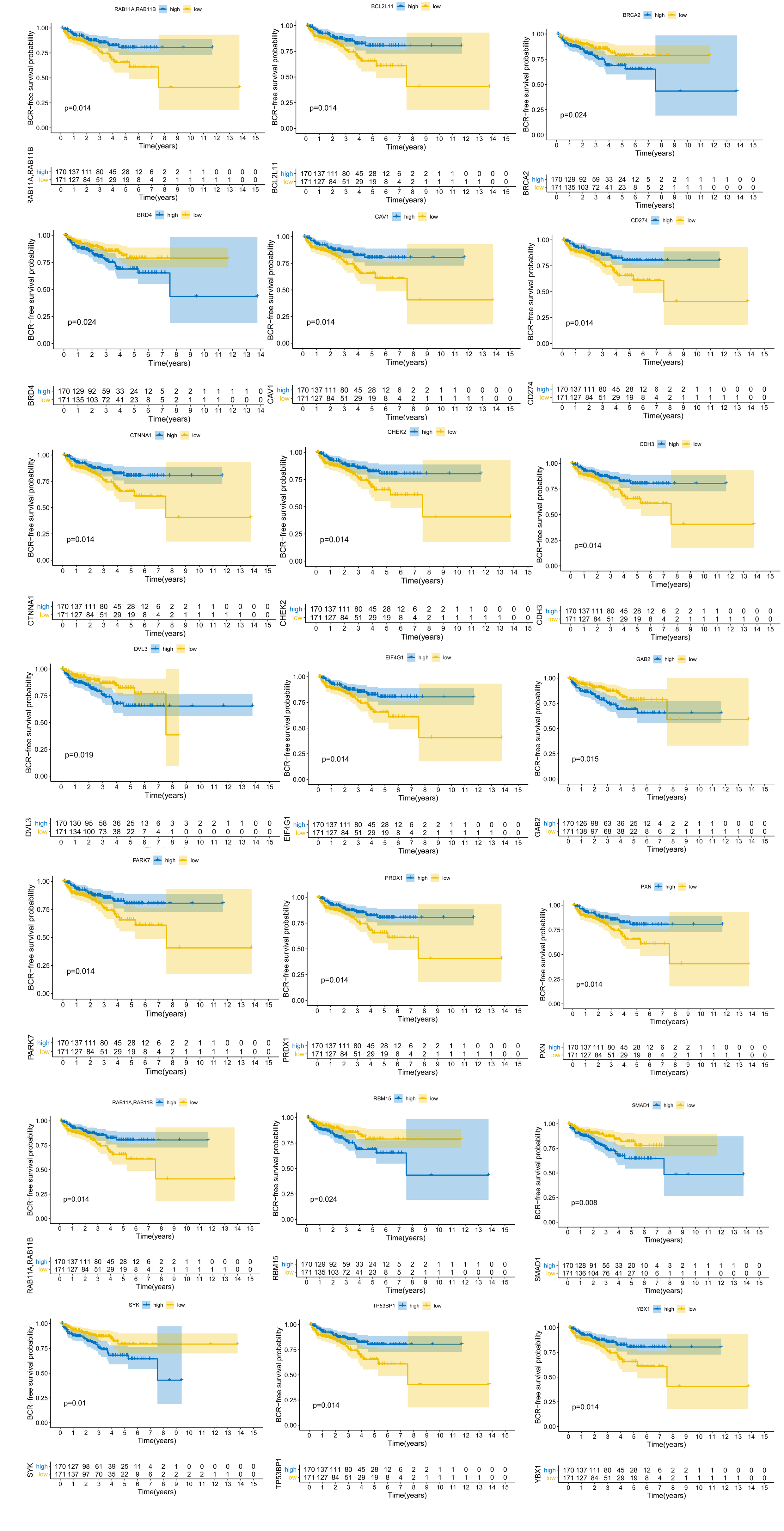

Supplement: Supplementary file 2 [file Image_2.TIF]

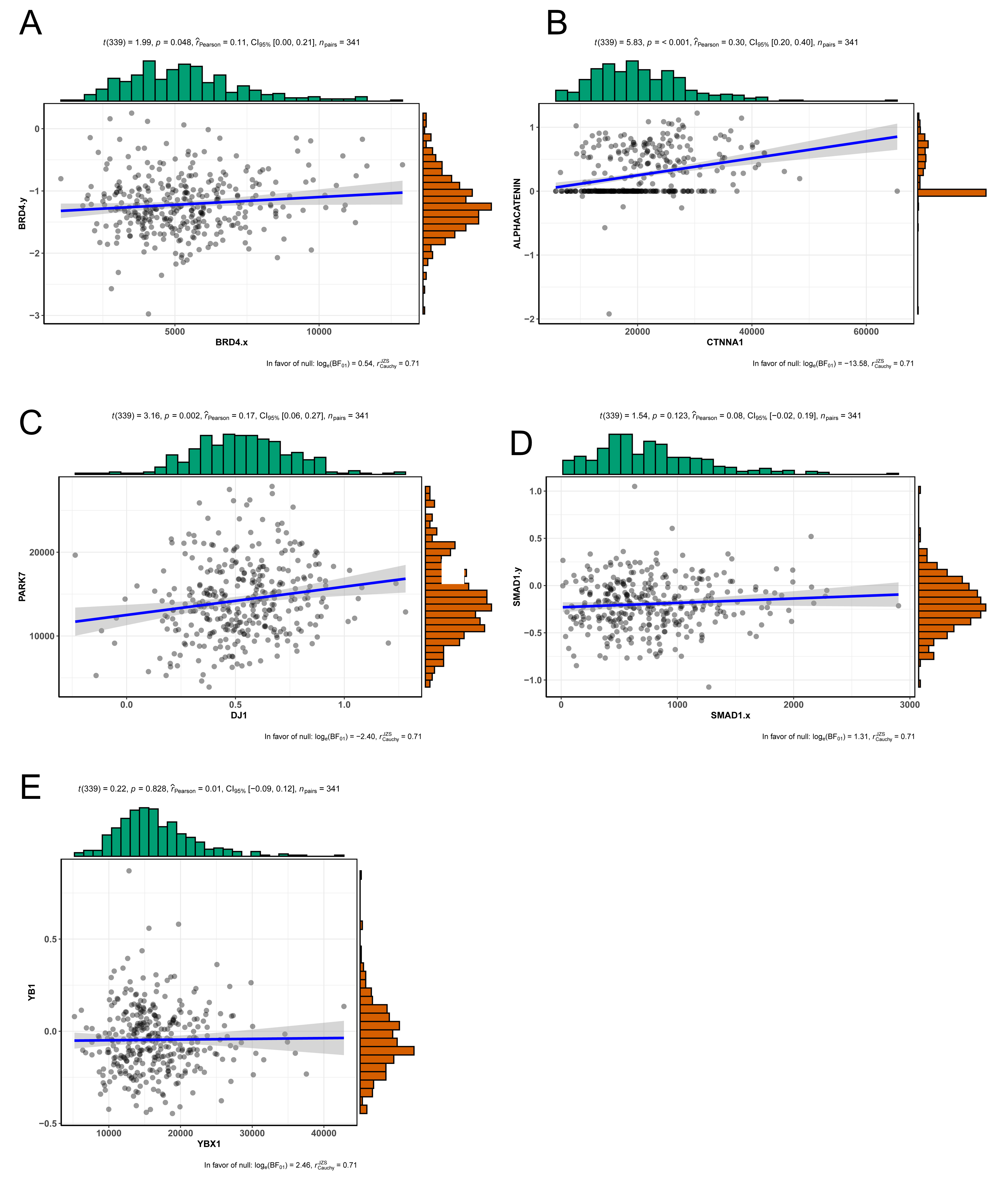

Supplement: Supplementary file 3 [file Image_3.TIF]
